# Association between Alcohol Intake and Prostate Cancer Mortality and Survival

**DOI:** 10.3390/nu15040925

**Published:** 2023-02-12

**Authors:** Oriana D’Ecclesiis, Elisa Pastore, Sara Gandini, Saverio Caini, Giulia Marvaso, Barbara A. Jereczek-Fossa, Giulia Corrao, Sara Raimondi, Federica Bellerba, Silvia Ciceri, Marialetizia Latella, Nora de Bonfioli Cavalcabò, Benedetta Bendinelli, Calogero Saieva, Miriam Fontana, Patrizia Gnagnarella

**Affiliations:** 1 Department of Experimental Oncology, European Institute of Oncology, IRCCS, 20139 Milan, Italy; 2 Institute for Cancer Research, Prevention, and Clinical Network (ISPRO), Via Cosimo il Vecchio 2, 50139 Florence, Italy; 3 Department of Oncology and Hemato-Oncology, University of Milan, 20122 Milan, Italy; 4 Division of Radiation Oncology, European Institute of Oncology, IRCCS, 20141 Milan, Italy; 5 Division of Epidemiology and Biostatistics, European Institute of Oncology, IRCCS, 20141 Milan, Italy

**Keywords:** alcohol, prostate cancer, mortality, survival, Meta-analysis

## Abstract

We conducted a systematic review and meta-analysis to investigate the role of alcohol consumption with the prognosis of prostate cancer (PCa). Published reports were gathered on 15 October 2022, from PUBMED/MEDLINE and EMBASE. We found 19 independent eligible studies on the association between consumption of alcoholic beverages and the risk of fatal PCa (n = 5), PCa mortality (n = 5) in healthy subjects, and PCa patients’ survival (n = 7) or surrogates thereof (n = 2). We used random effects meta-analysis to obtain a summary risk estimate (SRE) and 95% confidence intervals (95%CI) for incidence of fatal PCa and PCa mortality. The meta-analysis revealed no association between alcohol consumption and fatal prostate cancer incidence risk in healthy subjects with an indication for publication bias, but omitting the study that mainly increased the between-study heterogeneity, the SRE becomes significant (SRE 1.33, 95%CI 1.12–1.58), and the heterogeneity disappeared (*I*^2^ = 0%) with no indication of publication bias. No association of alcohol consumption was found with mortality risk in PCa patients (SRE 0.97, 95%CI 0.92–1.03) and PCa mortality risk in healthy subjects (SRE 1.03, 95%CI 0.82–1.30). In conclusion, this study suggests that there is some evidence of an association between high alcohol consumption and an increased risk of incidence of fatal prostate cancer in healthy subjects. Given the inconsistencies this result warrants further confirmation.

## 1. Introduction

Alcohol is considered as a carcinogen in humans by the Agency for Research on Cancer [1] and represents one of the top ten risk factors contributing to global cancer burden, together with other behavioral factors, including tobacco use, unsafe sex, and some dietary components. Specifically, about 4.9% of all male cancer deaths that occurred in 2019 were estimated to be attributable to alcohol consumption, as well as about 7.4% of all male cancer disability-adjusted life-years [2]. Moreover, according to the last report from the World Cancer Research Fund [3], there is strong evidence that alcoholic drinks consumption is a convincing or probable cause of several types of cancers, including cancers of the mouth, pharynx and larynx, esophagus (squamous cell carcinoma), breast (pre and post-menopause), colon–rectum, stomach, and liver. The risk of cancer is essentially due to ethanol intake, and, in general, tends to increase with the amount of ethanol consumed, irrespective of the type of alcoholic beverage [3].

Prostate cancer (PCa) is the second most common cancer among men worldwide and it ranks fifth in terms of mortality, as it was estimated to have caused over 375,000 deaths in 2020 [4]. Concerning the relationship of PCa with alcohol intake, the evidence is still inconclusive [5]. Indeed, studies reported mixed results: some studies found positive associations between alcohol consumption and PCa risk, also for moderate alcohol intake [6,7], while other studies found no association [8,9,10,11] or even an inverse association [12,13]. Overall, the two most recent meta-analyses of case-control and/or cohort studies [14,15] (which included, respectively, 27 and 11 studies) detected a positive association between PCa risk and alcohol intake, although some uncertainties persist because of inconsistency across studies in terms of results for aggressive vs. non-aggressive PCa, different alcoholic beverages (wine, beer, and liqueurs/spirits), and regarding the shape of the dose–response relationship (linear or non-linear). Our study, unlike the existing ones, besides being the most up-to-date, focuses on assessing the association between alcohol consumption and survival/mortality (and related outcomes) from PCa, whereas the two mentioned in the previous lines focus on alcohol consumption and PCa risk.

Alcohol consumption has also been investigated in relation to its possible effect on cancer mortality and the prognosis of cancer patients. To date, evidence on the relationship between alcohol consumption and survival or mortality in cancer patients, and particularly in PCa patients, is limited. In an ecological study, Penuelas and colleagues found a significant and positive association between per capita intakes of alcohol and mortality from total, colon, lung, breast, and prostate cancers [16]. In another study, more restrictive state alcohol policies were associated with lower mortality rates for six cancer types (esophagus, larynx, liver, oropharynx, prostate in male only, and breast in female only) among men and women in the US [17]. Evidence from epidemiological (non-ecological) studies is sparser: recently, a cohort study in South Korea showed that alcohol consumption was linearly and positively associated with cancer mortality, already among light drinkers [18], but other studies report conflicting results.

Despite the recognized detrimental effects of ethanol for human health and its known effect on morbidity and mortality [19], alcohol consumption is frequent in the healthy population [3] and even among cancer patients. Indeed, according to data from the 2012–2017 National Health Interview Survey (NHIS), nearly half (47.1%) of PCa patients were found to be light/moderate drinkers (up to 14 drinks per week), followed by a small proportion (4.2%) of heavy drinkers (>14 drinks per week) [20]. Given that low-volume drinking is very common among both healthy people and cancer patients, even a modest increase on cancer mortality and survival may have considerable clinical impact and public health implications, especially in the case of frequent diseases such as PCa. In light of these considerations, we believe it is important to provide a comprehensive picture regarding the effect of alcohol intake on prostate cancer-related outcomes. Accordingly, we conducted a systematic review and meta-analysis of all published articles that reported on the association between alcohol intake and PCa mortality (or incidence of fatal PCa) in healthy subjects and survival (or surrogates thereof) of PCa patients, in order to update the existing information and support public health policies on alcohol consumption in the general population and recommendations on the management of alcohol consumption in PCa patients.

## 2. Materials and Methods

The protocol of this systematic review is available online in the Open Science Framework (OSF) registry website (https://osf.io/2rvkq/) accessed on 28 December 2022).

### 2.1. Sources of Information and Search Strategy

A systematic literature search was conducted according to the PRISMA guidelines (Tables S1 and S10) [21]. Published reports were gathered on 15 October 2022, from PUBMED/MEDLINE and EMBASE. The publications were retrieved using the following search string: (alcohol* OR wine OR beer) AND (prostat*) AND (cancer OR malignanc* OR tumor OR tumour OR carcinoma) AND (survival OR outcome OR mortality OR prognos* OR progress*). No restrictions were applied, provided that an English abstract was available to make a preliminary decision about eligibility. The database search was supplemented by means of forward citation chaining, i.e., by perusing the reference list of eligible papers as well as previously published reviews and meta-analysis.

### 2.2. Eligibility Criteria, Articles Selection, and Data Extraction

The selection of articles for inclusion was conducted according to the PECO (Populations, Exposure, Comparison, Outcome) criteria illustrated in Table S2.

Concerning the exposure, we considered: (1) intake of alcoholic beverages (any, or separately for wine, beer, and others e.g., liquors/spirits), either categorized (e.g., either ever/never, current/former/never, etc.) or quantified in terms of drinking frequency (e.g., days/week); (2) alcohol intake (recent, cumulative/lifetime, or post diagnosis), either categorized (e.g., abstainers and light, moderate, and heavy drinkers) or quantified in terms of frequency (e.g., drinks/day) or actual amount (e.g., grams/day); (3) history of alcohol abuse (variously reported as alcoholism, alcohol dependence syndrome, etc.).

For estimates included in the meta-analysis, we converted the alcohol consumption categories for comparison in grams per day. We used information reported in the articles, and if missing data occurred, we consider one drink defined as one 12-fluid-ounce beer, one 5-ounce glass of wine, or one 1.5-ounce shot of liquor, all equaling approximately 13 g of alcohol [22].

Mendelian randomization studies considering genetically predicted alcohol intake or variants in genes involved in alcohol metabolism as the exposure of interest were also considered as eligible for inclusion in the review, although they were not entered in meta-analysis models.

In terms of outcome, we included papers that reported on any of (1) incidence of fatal PCa (i.e., of PCa eventually causing death) among healthy subjects; (2) PCa mortality (i.e., risk of death from PCa among healthy subjects); (3) overall and cause-specific survival of PCa patients, or (4) clinical surrogates thereof, e.g., post-diagnosis changes in PSA level including biochemical failure [23].

Upon removing duplicate items, all papers were initially screened out based on the title an abstract, and those that were retained were read in full text in order to decide on their eligibility. The selection of articles was conducted independently by two authors (SC and EP), and any disagreement was resolved by consensus with a third author (MF). The following information was extracted by two researchers (SG and OD) from each paper and entered in a dedicated spreadsheet: first author, year of publication, period of enrolment, country, study design, sample size, risk estimates and their 95% confidence intervals (CI; or another measure of statistical uncertainty, like standard errors or exact *p*-values, if those were not available), as well as the distribution of potential confounders of the studied association (e.g., patients’ demographics and tumor characteristics) and variables that were used for adjustment in statistical analyses. For the meta-analysis when more estimates were available, we considered the one regarding highest vs. lowest exposure and sensitivity analyses were conducted when the choice was not straightforward.

### 2.3. Quality Assessment

Given the exposure under study (alcohol consumption), all the studies included in the review have an observational design: therefore, the study quality and susceptibility to bias was assessed by using the Newcastle-Ottawa quality scale (NOS) [24] and the Quality in Prognosis Studies (QUIPS) tool [25]. In the NOS tool, a study can be assigned one star for each of the four and three items in the “Selection” and “Outcome” sections, and up to two stars for the item “Comparability”. The maximum score that a study can be assigned therefore equals nine stars, and studies are considered as good quality when the total score is six or above. Instead, a judgment of “low”, “moderate” or “high” risk of bias is given to each of the six domains of the QUIPS tool, namely “Study participation”, “Study attrition”, “Prognostic factor measurement”, “Outcome measurement”, “Study confounding”, and “Statistical analysis and reporting”. Interested readers can look into the documentation of either tool to learn more about the criteria that need to be applied to make the relevant quality judgments.

### 2.4. Statistical Analysis

The main characteristics of the studies included in the review were reported in tables and qualitatively summarized in narrative form. Meta-analysis was conducted by fitting random effect models, which output a summary risk estimate (SRE) and corresponding 95% CI for the association between high vs. low alcohol consumption. Risk estimates were pooled by means of meta-analysis for each outcome of interest for which we have at least three estimates with a similar exposure, i.e., risk estimates for alcoholism and from mendelian randomization were not pooled with the other estimates.

When more estimates from a single study had to be included, a hierarchical model was adopted to take into account that the estimates were not independent.

Statistical heterogeneity was evaluated through the I^2^ index and the Chi-squared test. The *I^2^* index can be interpreted as a measure of the percentage of the total variability of risk estimates across studies that is accounted for by actual heterogeneity instead of mere chance; as a thumb of rule, values of *I^2^* exceeding 50% are considered as an indicator of large between-study heterogeneity. Even when heterogeneity was large, however, subgroup analyses and/or meta-regressions could not be conducted due to the low number of included studies. We carried out sensitivity analyses in order to investigate whether between-study heterogeneity was due to single studies and whether conclusions depended on the choice made among the available estimates.

Possible publication bias was evaluated by means of Egger’s linear regression and Begg’s correlation test [26,27]. Furthermore, the possible presence of publication bias was assessed through visual inspection of the funnel plot, and exploratory analyzes were performed using trim and/or fill analysis in order to investigate and adjust the summary hazard ratio (SHR) estimate [28]. In particular, this method uses asymmetry to add possible unpublished studies and provide a revised summary estimate as a sensitivity analysis.

All reported *p* values were two sided and *p* < 0.05 was considered statistically significant. Meta-analyses were carried out using the Rstudio software (R version 4.1.1 R Core Team (2020). R: A language and environment for statistical computing. R Foundation for Statistical Computing, Vienna, Austria)).

## 3. Results

### 3.1. Characteristics of Eligible Studies

**Figure 1:**
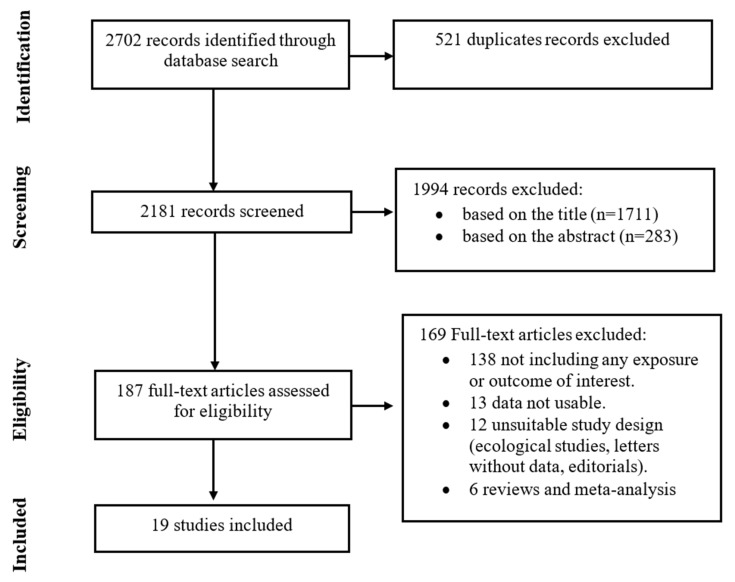
Flow-chart depicting the literature search and article selection for the systematic review of papers focusing on the association between alcohol intake and fatal prostate cancer risk, prostate cancer mortality, and survival (or surrogates thereof) of prostate cancer patients.

The literature search in PUBMED and EMBASE returned 2702 items, which dropped to 2181 after duplicates were removed (Figure 1). A total of 1994 articles were removed based on their title and abstract, which left 187 papers to be read in full text. Of these, an additional 169 were removed for not matching inclusion criteria, while there were 19 independent studies that were eligible and were included in the review for reporting on the association between consumption of alcoholic beverages and the risk of fatal PCa (n = 5) [22,29,30,31,32], PCa mortality (n = 5) [12,33,34,35,36], and PCa patients’ survival (n = 7) [13,37,38,39,40,41,42] or surrogates thereof (n = 2) [43,44].

The main results and study characteristics of the selected studies are presented in Table 1, Table 2, Table 3 and Table 4 and Supplementary Tables S3–S6, respectively. Of the included studies, the majority are prospective studies (46.7%) and the most frequent study design is the cohort-study (80%). Among the 19 studies included, 11 were carried out in North America [13,20,29,30,34,37,38,39,41,42,43], 4 in Europe [31,32,36,44], 2 in Asia [33,35], 1 in Australia [12] and 1 included data from 25 studies within the international PRACTICAL Consortium (USA, Australia and European countries) [40]. Two studies were large-scale prospective study involving more than 1,300,000 men conducted in Korea [33] and in Sweden [32]. The statistical analysis most commonly used in the included studies is the Cox proportional hazards regression model for survival data.

### 3.2. Fatal Prostate Cancer Incidence in Healthy Subjects

**Figure 2:**
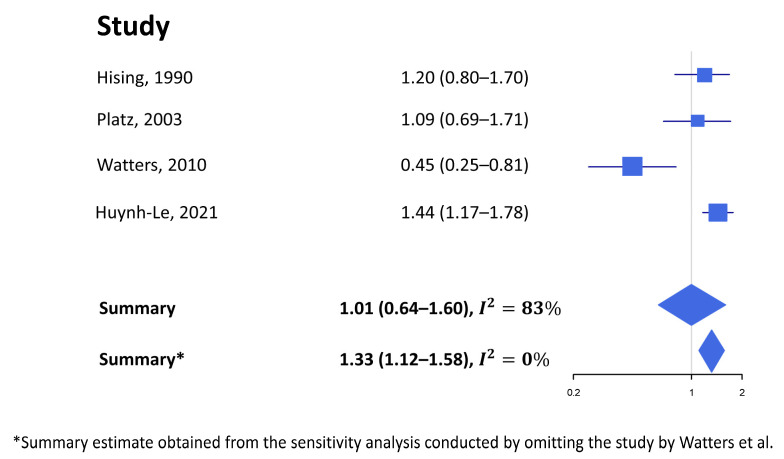
Forest plot of the measures of association between alcohol intake (highest vs. lowest category) and the risk of fatal prostate cancer [22,29,30,31].

Five studies reported on the association between alcohol intake and the risk of fatal PCa (Table 1), of which four had a cohort design, while the study by Huynh-Le et al. was a nested case-control study [31]. The latter was the only study to show a significant association between alcohol consumption and fatal PCa risk: the RR was 1.44 (95% CI 1.17–1.78 for those with vs. without a history of alcohol intake). The meta-analysis including four papers, revealed no association between alcohol consumption (highest vs. lowest) and fatal PCa incidence risk in healthy subjects (SRE 1.01, 95% CI 0.64–1.60, Figure 2) [22,29,30,31]. Dahlman 2022 was not included because the risk estimate is for alcohol abuse/alcoholism [32].

Although Egger’s regression showed that there was publication bias (*p*-value 0.007), Begg’s test (*p*-value 0.08) and the trim and fill analysis did not detect any potentially missing studies as no asymmetry was revealed from the funnel plot.

The sensitivity analysis showed that the large heterogeneity (I^2^ 83%) was entirely attributable to the study by Watters et al. [22], in which a significant inverse association between alcohol intake and fatal PCa risk was found for heavy drinkers. Upon omitting this study from the meta-analysis, the SRE becomes significant (SRE 1.33, 95% CI 1.12–1.58, Figure 2), the heterogeneity disappeared (*I^2^* 0%), and there was no indication of publication bias according to both Egger’s regression (*p*-value 0.21) and Begg’s test (*p*-value 0.33). Finally, the results did not change by replacing the risk estimate for beer consumption with that for liquors in Hsing’s study [29]. The meta-regression showed that study quality is not an effect modifier (*p* = 0.13).

### 3.3. Prostate Cancer Mortality in Healthy Subjects

The literature search identified five independent studies examining the relationship between alcohol intake and the risk of death from PCa (Table 2) in healthy subjects: these included four studies with a prospective cohort design [12,33,34,36], while the study by Fowke et al. reported results from the pooled analysis of 18 prospective cohort studies included in the Asia Cohort Consortium [35]. Overall, evidence of an association of alcohol intake with PCa-specific mortality is scant. Breslow et al. found that the risk was increased among men drinking alcohol beverages 1–2 or ≥3 days per week compared to those drinking alcohol less than once weekly (the HR were 1.70, 95% CI 1.09–2.64, and 1.55, 95% CI 1.01–2.38, respectively), but similar analyses focusing on the amount of alcohol (instead of frequency of consumption) did not yield any significant results [34]. The only significant result went in the opposite direction in the study by Dickermann et al., where abstainers compared to light drinkers were found to have a higher risk of death from PCa (HR 1.90, 95% CI 1.04–3.47) [36]. In the studies by Baglietto et al., Kim et al., and Fowke et al., alcohol did not appear to be significantly associated with PCa mortality (Table 2) [12,33,35].

**Figure 3:**
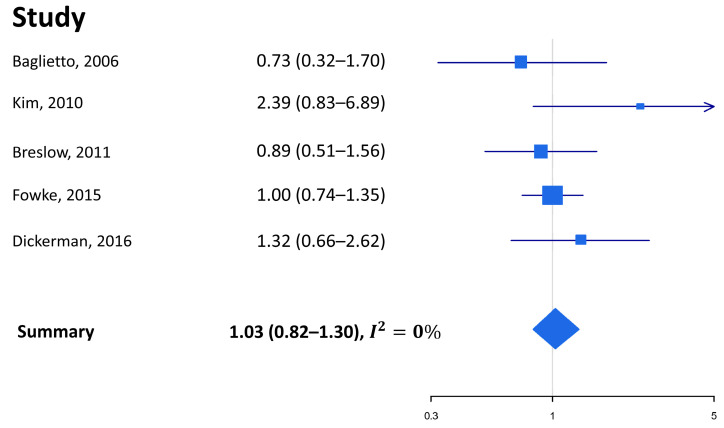
Forest plot of the measures of association between alcohol intake (highest vs. lowest category) and prostate cancer mortality [12,33,34,35,36].

The meta-analysis comparing the highest vs. lowest category of alcohol consumption confirmed the lack of an association between alcohol consumption and PCa mortality risk in healthy subjects (SRE 1.03, 95% CI 0.82–1.30, Figure 3), with data being highly consistent across studies (I^2^ 0%). There was no indication that publication bias was at play (Begg’s *p*-value 0.82, Egger’s *p*-value 0.43), and the trim and fill method also confirmed that there were no potentially missing studies as no asymmetry was revealed from the funnel plot. The meta-regression showed that study quality is not an effect modifier (*p* = 0.97).

### 3.4. Survival (and Surrogates Thereof) among Prostate Cancer Patients

We identified seven studies that prospectively investigated the association between alcohol consumption and survival among PCa patients (Table 3) [13,37,38,39,40,41,42]. The main source of heterogeneity by design was in the way the exposure of interest was defined and measured, since the included studies quantified the risk of all-cause and cause-specific death of PCa patients in relation to exposures as ever vs. never use of alcohol, post-PCa diagnosis alcohol intake, alcoholism / alcohol dependence syndrome, or the presence of variants in alcohol-metabolizing genes (in the Mendelian randomization study by Brunner et al. [40]).

Similarly, to PCa mortality, also concerning the survival of PCa patients only a minority of studies reported a significant association with alcohol intake. The studies by Yu et al., Bluethmann et al., and Downer et al. did not detect any effect of alcohol intake on PCa patients’ survival [13,37,42]. Those with a history of alcoholism had a significantly shorter overall survival (OS) in the study by Chamie et al. (HR 1.77, 95% CI 1.07–2.93) [38], which was confirmed in the study by Jayadevappa et al., although limited to patients aged 66–74 years (HR 1.4, 95% CI 1.1–1.8) [39]. In the study by Farris et al., there was some suggestion that the frequency of post-diagnosis alcohol intake may instead reduce the risk of all-cause death, although the trend was non-linear and statistical significance was only achieved in one category of intake (drinks/week >0.9 to <3.7 vs. none) [41]. Finally, the Mendelian randomization study by Brunner et al. found an association between the rs10973794 variant in the ALDH1B1 gene and a shorter cause-specific survival among patients with low grade PCa (HR 1.43, 95% CI 1.14–1.79) [40].

**Figure 4:**
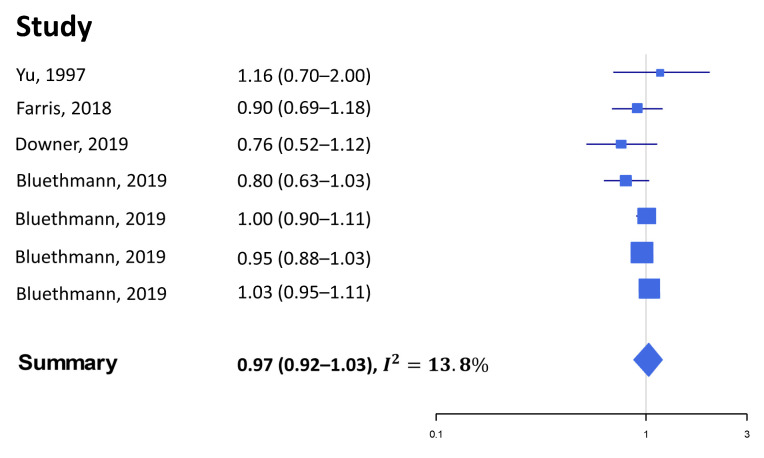
Forest plot of the measures of association between alcohol intake (highest vs. lowest category) and the overall survival of prostate cancer patients [13,37,41,42]. The four estimates from Bluethmann refer to four age groups in the following order: 40–54; 55–64; 65–74; 75+.

The meta-analysis showed that there was no association between alcohol consumption and mortality risk in prostate cancer patients (SHR 0.97, 95% CI 0.92–1.03, Figure 4). 

No publication bias was reported (Begg’s *p*-value 0.77 and Egger’s *p*-value 0.19). The heterogeneity across studies was not large (*I*^2^ 13.8). Using the trim and fill method, potentially missing studies were found, since the funnel plot revealed asymmetry, but did not change the results (SHR 0.99, 95% CI 0.94–1.04).

Finally, two studies examined PCa patients’ alcohol intake in relation to clinical parameters that may indicate cancer progression and are known to be associated with survival, as postdiagnosis increases in PSA levels with time (PSA growth). Ly et al., reported occurrence of biochemical failure and Burton et al. the growth of PSA concentration (Table 4). In both studies, alcohol intake was not found to be associated with the corresponding surrogate of PCa patients’ survival [43,44]. All studies included in this scenario except one (Yu et al.) had a low risk of bias.

Alcohol consumption categories for comparison in grams per day for estimates included in the meta-analysis are presented in Table S7.

### 3.5. Quality Assessment and Susceptibility to Bias

The study quality was generally fair (Supplementary Tables S8 and S9); the main limitations were the adequacy of follow-up, adjustment for important confounders such as the ones related to socio-economic status and consistent ascertainment of exposure.

In particular, with regard to the incidence of fatal prostate cancer scenario, the article with the lowest quality score is Huynh Le et al. The latter lacks adequate cohort follow-up, good outcome assessment, exposure assessment and does not make clear whether the outcome of interest was absent at the start of the study. On the other hand, studies on prostate cancer mortality show high quality scores. Among studies reporting surrogates of prostate cancer patient survival: the study by Ly et al. is at high risk of bias due to possible confounding factors not considered in the statistical analysis. While in studies reporting the overall and cancer-specific survival of prostate cancer patients, the works showing the greatest risk of bias are those of Yu et al. and Brunner et al.

## 4. Discussion

In this systematic review and meta-analysis overall, we found no significant association between alcohol consumption and incidence of fatal prostate cancer (five studies) or prostate cancer mortality (five studies) in healthy subjects. In studies conducted among PCa patients, our meta-analysis showed that there was no association between alcohol consumption and overall or cancer-specific survival (seven studies). Finally, both studies that examined PCa patients’ alcohol intake in relation to clinical parameters known to correlate with survival, found no association with the corresponding surrogate of survival. Indeed, the results of individual studies included in our review are mixed and inconsistent.

Regarding the relationship between alcohol consumption and fatal PCa incidence, only one study [31] included in our review detected a significant association, which was found between history of alcohol intake and increased fatal disease risk in a cohort of Swedish men. However, summary risk estimate of the meta-analysis becomes significant upon omitting the study by Watters et al., which was found to be responsible for the largest share of heterogeneity among studies [22]. This result is consistent with some evidence in the literature of a direct association between alcohol consumption and PCa risk, which emerged in the studies by Sesso et al. [6] and Putnam et al. [7]. It is interesting to note that Watters is the only study that suggested a protective effect of heavy drinking in fatal cancers. The authors analyzed the data collected in the NIH–AARP Diet and Health Study, a large prospective cohort involving 294,707 US men. High alcohol exposures could alter hormonal profiles and lower circulating testosterone, explaining the protective association [45].

With respect to PCa mortality among healthy subjects, two studies included in our review reported a significant association with alcohol consumption, but with opposite results: Breslow et al. found an increased risk of PCa mortality among higher-frequency drinkers (1–2 or ≥3 days per week vs. less than once weekly), while the study by Dickerman et al. showed that abstainers had a greater risk of death from PCa compared to light drinkers (0.01–3 drinks/week) [34,36]. Dickerman collected information on alcohol consumption using a questionnaire administered two times, in 1975 and 1981 distinguishing lifetime abstainers from former drinkers. The latter was consistent with the results by Farris et al. that assessed the frequency of post-diagnosis alcohol intake in relation to PCa survival in a cohort of Canadian patients and reported a significant protective association between low alcohol consumption (>0.9 to <3.7 drinks/week) and all-cause mortality [41]. However, it must be emphasized that, in this study, the reference category (non-drinkers) included the participants who did not consume alcohol in the time period of data collection, without distinguishing between lifetime abstainers and former drinkers and so generating a potential bias. Accurate measurement of alcohol exposure is difficult in terms of quantity and frequency [46]. It is usually assessed once at baseline through food frequency questionnaires that do not properly distinguish lifetime consumption, e.g., former drinkers from infrequent drinkers or abstainers, who may have very different risk profiles. The misclassification of former drinkers as abstainers is a problem often discussed in the literature; for example, in the meta-analysis conducted by Zhao et al., the association between different levels of alcohol exposure and increased risk of PCa was stronger in the studies free from the aforementioned misclassification error, which may therefore have led to an underestimation of the risks [14]. Since prostate tumors are typically slow-growing and the biological mechanisms by which alcohol might influence its progression are not yet fully elucidated, it might be useful in all future studies to separate lifelong abstainers from those who have stopped or reduced alcohol consumption (also taking into account the reasons for the reduction/cessation) in order to get more accurate results.

Moreover, with regard to alcohol consumption among PCa patients, two studies included in our review [38,39] found that the participants with a history of alcoholism had a significantly shorter OS. These results are consistent with those reported in the study by Saieva et al., who observed an increase in total mortality and in a series of other death causes, including cancer, in a cohort of Italian alcoholics, in comparison with the general population of the area [47]. However, it must be emphasized that alcohol abuse is often associated with other addictions (e.g., smoking) and diseases (e.g., psychiatric illnesses), that can affect lifestyle, cancer risk and survival, so that controlling for confounding is practically very challenging and unlikely to be fully attained. Nevertheless, in the most recent review on alcohol and PCa, conducted by Macke and Petrosyan, the authors concluded that high alcohol intake, especially binge drinking, was associated with increased PCa risk and that alcohol consumption was directly linked to PCa mortality as it may accelerate the growth of prostate tumors [48].

Our study has some limitations that are appropriate to mention. First, the validity of our results is curbed by the low number of available studies for each of the outcomes under scrutiny, by their methodological limitations, and above all by their heterogeneity. The main source of heterogeneity lies in the way the exposure of interest was defined and measured, since the included studies quantified the risk of death from all causes and from specific causes in healthy subjects or PCa patients in relation to different exposures such as lifetime (or never) alcohol use, alcohol intake after PCa diagnosis, alcoholism/alcohol dependence syndrome, or the presence of variants in alcohol metabolizing genes. In addition, as we have already mentioned, there may be misclassification errors in the reference categories. For example, sicker men who had drunk in the past and stopped drinking at baseline, could be misclassified attenuating the association.

In this meta-analysis we could not study dose–response relationships of alcohol and prostate cancer outcomes due to missing information. Subsequent research should investigate this aspect.

The lack of association reported by studies can be due to the possible effect of uncontrolled confounding factors such as diabetes history. The use of antidiabetic medications such as metformin, can exert a protective effect, reducing risk of prostate cancer as reported in the literature [49,50]. The results were also heterogeneously reported in terms of alcohol consumption. In general, total alcohol consumption is estimated but the categories used for comparison are heterogenous, with a very wide range of highest alcohol consumption (from 0.2 to ≥90 g/day) not associated with any geographical region. Available tools are limited by ability to measure initiation and maintenance of patterns of alcohol consumption over time in populations and their subgroups and methodological studies are needed to refine instruments that will improve self-reported ethanol intake [46]. Well conducted studies with proper study designs should further confirm the role of diet and lifestyle, including alcohol consumption, in prostate prognosis [51].

## 5. Conclusions

In conclusion, this systematic review and meta-analysis indicates that there is some evidence that alcohol consumption may play a role in fatal prostate cancer risk. Alcohol consumption has been linked to an increased likelihood of developing over 200 health conditions, not only cancers and cardiovascular diseases, but also liver diseases, road injuries and violence, suicides, tuberculosis and HIV/AIDS [52]. For the other outcomes, no significant association with alcohol consumption was found, but this may be due to misclassification of exposures. Furthermore, the relation between alcohol consumption and health outcomes is complex and multidimensional. To date, no studies have shown any potential protective effect of alcohol and there is no evidence of a particular threshold at which alcohol starts to manifest its carcinogenic effects. Within actions and recommended interventions to reduce the harmful use of alcohol proposed by WHO [52], future studies are warranted to clarify heterogeneous findings and associations between alcohol consumption and fatal PCa and/or PCa mortality. The epidemiology of alcohol use plays a vital role not only in PCa research, but it has significant social and economic consequences.

## Figures and Tables

**Table 1:** Summary results of the studies included in the review that reported on the association between alcohol intake and the risk of fatal prostate cancer.

First Author, PY	Exposure	RR (95%CI)	Adj
Hsing A.W. et al. 1990 [29]			Age
	Beer		
	Former vs. never	1.7 (1.0–2.9)	
	Current vs. never	1.2 (0.8–1.7)	
	Liquor		
	Former vs. never	0.7 (0.3–1.5)	
	Current vs. never	1.0 (0.7–1.4)	
Platz A.E. et al. 2003 [30]			Current age, BMI at age 21, height, smoking, FHPC, major ancestry, diabetes, vasectomy, vigorous PA, intakes of total energy, calcium, fructose, tomato sauce, red meat, fish, vitamin E and α-linolenic acid
	Drinking frequency (days/week)	Distant metastatic or fatal cases	
	1–2 vs. 0 ^1^	0.83 (0.59–1.18)	
	3–4 vs. 0 ^1^	1.00 (0.67–1.51)	
	5–6 vs. 0 ^1^	1.18 (0.80–1.75)	
	7 vs. 0 ^1^	0.76 (0.51–1.14)	
	Alcohol intake (g/day)	Distant metastatic or fatal cases	
	0.1–4.9 vs. 0 ^1^	0.83 (0.57–1.21)	
	5.0–14.9 vs. 0 ^1^	1.09 (0.78–1.57)	
	15.0–29.9 vs. 0 ^1^	0.85 (0.53–1.35)	
	30.0 −49.9 vs. 0 ^1^	1.09 (0.69–1.71)	
Watters L.J. et al. 2010 [22]			Age, race, education, marital status, height, BMI, PA, FHPC, diabetes, self-reported health status, smoking, PSA screening, digital rectal examination, total energy (excluding alcohol), α-tocopherol, calcium, red meat, fish, tomato, α-linolenic acid, selenium
	Alcohol (Drinks/Day)	Fatal cases	
	<1 vs. 0 ^1^	0.86 (0.69–1.08)	
	1–3 vs. 0 ^1^	0.95 (0.72–1.26)	
	>3 and <6 vs. 0 ^1^	0.81 (0.53–1.21)	
	≥6 vs. 0 ^1^	0.45 (0.25–0.81)	
Huynh-Le M.P. et al. 2021 [31]			FHPC, Diabetes history, age
	history of alcohol intake	1.44 (1.17–1.78) ^2^	
	Yes vs. no ^1^		
	alcohol intake with the polygenic hazard score (PHS46):	1.45 (1.19–1.76) ^2^	FHPC, Diabetes history, age, PHS46
	Yes vs. no ^1^		
	alcohol intake with the polygenic hazard score (PHS166)	1.52 (1.22–1.88) ^2^	FHPC, Diabetes history, age, PHS166
	Yes vs. no ^1^		
Dahlman D. et al. 2022 [32]	Alcohol use disorder	0.90 (0.82–0.97)	age, educational attainment, social welfare, marital status, region of residence, immigrant status, comorbidities

^1^ No clear distinction between lifetime abstainers and former drinkers. ^2^ Hazard Ratio. P: Poisson regression; C: Cox proportional hazards regression models. P. Prospective. CC. case-control.

**Table 2:** Summary results of the studies included in the review that reported on the association between alcohol intake and the risk of death from prostate cancer in healthy subjects.

First Author, PY	Exposure	RR (95%CI)	Adj
Baglietto L. et al. 2006 [12]	Total alcohol (g/day)		Country of birth
	1–19 vs. Lifetime abstainers	0.56 (0.28,1.14)	
	20–39 vs. Lifetime abstainers	0.60 (0.26,1.38)	
	40+ vs. Lifetime abstainers	0.73 (0.32,1.70)	
Kim M.K. et al. 2010 [33]	Alcohol consumption, g/day:		Age, residential, smoking, regular exercise, BMI, systolic and diastolic blood pressure, fasting blood sugar
	1.0–14.9 vs. Non drinker ^1^	1.32 (0.59–3.00) ^2^	
	15–29.9 vs. Non drinker ^1^	1.75 (0.72–4.22) ^2^	
	30–89.9 vs. Non drinker ^1^	2.09 (0.84–5.19) ^2^	
	≥90 vs. Non drinker ^1^	2.39 (0.83–6.89) ^2^	
Breslow R.A. et al. 2011 [34]	Former drinker ^3^ vs. Never drinker ^4^	1.12 (0.81–1.55)	Race/ethnicity, education, region, marital status, smoking status, BMI
	Lifetime infrequent drinker ^5^ vs. Never drinker	1.00 (0.67–1.48)	
	Current drinker ^6^:		
	Light ^7^ vs. Never drinker	0.93 (0.66–1.30)	
	Moderate ^8^ vs. Never drinker	1.22 (0.86–1.72)	
	Heavier ^9^ vs. Never drinker	0.89 (0.51–1.56)	
	Among Current Drinkers (quantity)		
	Q = 2 vs. Q = 1	0.93 (0.61–1.41)	
	Q ≥ 3 vs. Q = 1	0.90 (0.58–1.39)	
	Frequency (average number of drinking days per week)		
	F = 1/2 vs. F < 1	1.70 (1.09–2.64)	
	F ≥ 3 vs. F < 1	1.55 (1.01–2.38)	
Fowke J.H. et al. 2015 [35]			Age, education, population density, marital status, history of severe cancer, heart disease or stroke at baseline
	Level of Alcohol Consumption		
	None ^1^ vs. 1–155 g/week	1.02 (0.78–1.34)	
	≥156 g/week vs. 1–155 g/week	1.00 (0.74–1.35)	
Dickerman B.A. et al. 2016 [36]	Alcohol consumption (continuous)	1.06 (0.91–1.23)	BMI, smoking, social class, education, PA
	Abstainers vs. light drinkers	1.90 (1.04–3.47)	
	Moderate vs. light drinkers	1.22 (0.76–1.97)	
	Heavy vs. light drinkers	1.32 (0.66–2.62)	
	Binge drinking status		
	Yes vs. no	0.87 (0.52–1.45)	

^1^ no clear distinction between lifetime abstainers and former drinkers. ^2^ RR: Relative risks (RRs) and 95% confidence intervals (95% CIs) for death from all-causes and from cancer according to alcohol consumption among men. ^3^ Former drinker: who consumed 12 or more drinks in their lifetime and 12 or more drinks in any previous year but no drinks in the past year. ^4^ Never drinkers: who consumed fewer than 12 drinks over the course of their lifetime. ^5^ Lifetime infrequent drinkers: who consumed 12 or more drinks in their lifetime but fewer than 12 drinks in any previous year. ^6^ Current drinker: who had consumed 1 or more drinks in the past year (12 or more drinks in the past year for 1988). ^7^ Light drinker: women ≤3 drinks per week, in men, ≤3 drinks per week; ^8^ Moderate drinker: women >3–7 drinks per week, men >3–14 drinks per week; ^9^ Heavier drinker: women >7 drinks per week; men >14 drinks per week.

**Table 3:** Summary results of the studies included in the review that reported on the association between alcohol intake and the overall and cancer-specific survival of prostate cancer patients.

First Author, PY	Exposure	HR (95%CI)	Adj
Yu G.P. et al. 1997 [37]	**Alcohol use**:		not specified
	Ever vs. never ^1^	1.16 (0.70–2.0)	
Chamie K. et al. 2012 [38]	**History of alcoholism**	Non-prostate Cancer-related Mortality	not specified
	Yes vs. no	1.77 (1.07–2.93) ^3^	
Jayadevappa R. et al. 2016 [39]			Race, ethnicity, marital status, census tract median income, census tract proportion with college education, geographic area, disease severity, co-morbidity, prostate cancer treatment
	**Type of substance use disorder:**		
	Alcohol dependence syndrome	Overall survival:	
	Age 66–74	1.4 (1.1–1.8)	
	Age ≥ 75	0.8 (0.6–1.2)	
Brunner C. et al. 2016 [40]	**SNPs within ALDH1A2 following a diagnosis of any prostate cancer:**	Cause-specific survival:	not specified
	rs1441817	0.78 (0.66–0.91) ^4^	
	rs12910509	0.76 (0.64–0.91) ^4^	
	rs8041922	0.76 (0.64–0.91) ^4^	
	**In ALDH1B1 following a diagnosis of low grade prostate cancer:**		
	rs10973794	1.43 (1.14–1.79) ^4^	
Farris M.S. et al. 2018 [41]			Age, stage, PSA levels, Gleason score, smoking status and BMI at diagnosis, prostatectomy, hormone therapy, Charlson comorbidity score, lifetime total physical activity, education level and how often (on average) participants went for a general check-up in their lifetime prior to diagnosis of prostate cancer
	**Post-diagnosis dose intake drinks/week**	Overall survival:	
	>0 to <0.9 vs. none ^1^	0.76 (0.58–1.00)	
	>0.9 to <3.7 vs. none ^1^	0.77 (0.61–0.98)	
	>3.7 to <8.3 vs. none ^1^	0.76 (0.58–1.01)	
	≥8.3 vs. none ^1^	0.90 (0.69–1.18)	
	**Post-diagnosis dose of alcohol intake (drinks/week)**	Cause specific mortality ^2^	
	>0 to <0.9 vs. none ^1^	0.93 (0.56–1.52)	
	>0.9 to <3.7 vs. none ^1^	1.06 (0.71–1.59)	
	>3.7 to <8.3 vs. none ^1^	0.89 (0.54–1.45)	
	≥8.3 vs. none ^1^	1.31 (0.85–2.04)	
	**Post-diagnosis dose of alcohol intake (drinks/week)**	Cause specific mortality ^3^	
	>0 to <0.9 vs. none ^1^	1.08 (0.65–1.78)	
	>0.9 to <3.7 vs. none ^1^	1.23 (0.80–1.87)	
	>3.7 to <8.3 vs. none ^1^	1.03 (0.63–1.70)	
	≥8.3 vs. none ^1^	1.47 (0.91–2.37)	
Bluethmann S.M. et al. 2019 [42]	Chronic drinking ^5^		Race/ethnicity, insurance status, rurality, lymph node status, PC aggressiveness, serum PSA and behavioral risk factors from BRFSS (smoking, obesity, physical inactivity, chronic drinking and FV intake)
	Subgroup by age:	Cause-specific survival:	
	40–54	0.80 (0.63–1.03)	
	55–64	1.00 (0.90–1.11)	
	65–74	0.95 (0.88–1.03)	
	75+	1.03 (0.95–1.11)	
Downer M.K. et al. 2019 [13]			Total energy intake, smoking, BMI. Vigorous physical activity, choline, coffee, lycopene, whole milk, diabetes, PSA screening,
	First post-diagnostic report intake g/d	Overall survival:	
	>0 to <10 vs. none ^1^	0.84 (0.64–1.10)	
	>10 to <15 vs. none ^1^	0.77 (0.54–1.10)	
	15 to <30 vs. none ^1^	0.71 (0.50–1.00)	
	≥30 vs. none ^1^	0.76 (0.52–1.12)	
		Cause specific survival:	
	>0 to <10 vs. none ^1^	1.17 (0.63–2.19)	
	>10 to <15 vs. none ^1^	1.02 (0.45–2.29)	
	15 to <30 vs. none ^1^	0.63 (0.31–1.31)	
	≥30 vs. none ^1^	1.32 (0.53–3.25)	

^1^ no clear distinction between lifetime abstainers and former drinkers. ^2^ Contributing risk Fine and Gray mode: Contributing risk is death from other causes, not specific prostate cancer. ^3^ Competing-risks regression analysis with failure defined as non-prostate cancer-related mortality and the competing risk as prostate cancer-specific Mortality: hazard of non-prostate cancer mortality. ^4^ Odds Ratio from mendelian randomization study. ^5^ Chronic use of alcohol/high risk for heavy drinking (defined as an average ≥2 drinks per day for the last 30 days vs. <2 drinks). In bold the estimates included in the meta-analyses.

**Table 4:** Summary results of the studies included in the review that reported on the association between alcohol intake and clinical surrogates of survival of prostate cancer patients.

First Author, PY	Exposure	HR (95%CI)	Adj
Ly D. et al. 2010 [43]	Alcohol consumption:	Biochemical failure: 1.15 (0.91–1.45)	No
	Yes vs. No		
Burton A.J. et al. 2012 [44]	Alcohol (per 10 units a week)	PSA at age 50 (expressed as % difference in baseline PSA):	
		−2.10 (−5.00–0.8) ^1^	Age, height, weight, WC, HC, inside leg, occupational class, smoking status, exercise
		−2.20 (−5.1–0.70) ^2^	Age, height, weight, WC, HC, inside leg, occupational class, smoking status, exercise, Gleason score
		Yearly increase in PSA (expressed as %change in yearly increase in PSA):	
		−0.20 (−0.40–0.10) ^1^	Age, height, weight, WC, HC, inside leg, occupational class, smoking status, exercise
		−0.20 (−0.40–0.10)	Age, height, weight, WC, HC, inside leg, occupational class, smoking status, exercise, Gleason score

^1^ All covariates included in the same model, accept BMI (due to collinearity with weight and height). ^2^ As ^1^ but additionally adjusted for Gleason score. Biochemical failure defined by Ly et al. as PSA increase of 2 ng/mL or greater from a posttreatment nadir for radiation modalities and PSA 0.4 ng/mL or higher after surgery.

## Data Availability

All relevant data are within the paper and its Supplementary Materials .

## Supplementary Materials

The following supporting information can be downloaded at: https://www.mdpi.com/article/10.3390/nu15040925/s1, Table S1. PRISMA checklist; Table S2. PECO (P = Population, E = Exposure, C = Comparison, O = Outcome) criteria for the systematic literature review on the association between alcohol intake and the incidence of fatal prostate cancer (PCa), PCa mortality, and the overall and cancer-specific survival, and clinical surrogates of survival, of PCa patients; Table S3: Main characteristics of the studies included in the review that reported on the association between alcohol intake and the risk of fatal prostate cancer; Table S4: Main characteristics of the studies included in the review that reported on the association between alcohol intake and the risk of death from prostate cancer in healthy subjects; Table S5: Main characteristics of the studies included in the review that reported on the association between alcohol intake and the overall and cancer-specific survival of prostate cancer patients; Table S6: Main characteristics of the studies included in the review that reported on the association between alcohol intake and clinical surrogates of survival of prostate cancer patients; Table S7: Estimated alcohol intake expressed in grams per day (g/day) for estimates and studies included in the meta-analysis; Table S8. Quality in Prognostic Studies (QUIPS) tool; Table S9. Newcastle-Ottawa quality assessment scale; Table S10. PRISMA 2020 for Abstracts.
