# Supplementary material for: Association between Alcohol Intake and Prostate Cancer Mortality and Survival
Source: Nutrients. 2023 Feb 12;15(4):925. doi: 10.3390/nu15040925 (PMC9965886; doi:10.3390/nu15040925)
Supplement: Supplementary file 1 [file nutrients-15-00925-s001.zip › nutrients-2164511-supplementary.pdf]

nutrients MDPI

Tables and Figures

Table S1. PRISMA checklist.

Section and Topic	Item #	Checklist item	Location where item is reported
**TITLE**			
Title	1	Identify the report as a systematic review.	1
**ABSTRACT**			
Abstract	2	See the PRISMA 2020 for Abstracts checklist.	S5
**INTRODUCTION**			
Rationale	3	Describe the rationale for the review in the context of existing knowledge.	2
Objectives	4	Provide an explicit statement of the objective(s) or question(s) the review addresses.	3
**METHODS**			
Eligibility criteria	5	Specify the inclusion and exclusion criteria for the review and how studies were grouped for the syntheses.	3
Information sources	6	Specify all databases, registers, websites, organisations, reference lists and other sources searched or consulted to identify studies. Specify the date when each source was last searched or consulted.	3
Search strategy	7	Present the full search strategies for all databases, registers and websites, including any filters and limits used.	3
Selection process	8	Specify the methods used to decide whether a study met the inclusion criteria of the review, including how many reviewers screened each record and each report retrieved, whether they worked independently, and if applicable, details of automation tools used in the process.	3-4
Data collection process	9	Specify the methods used to collect data from reports, including how many reviewers collected data from each report, whether they worked independently, any processes for obtaining or confirming data from study investigators, and if applicable, details of automation tools used in the process.	3-4
Data items	10a	List and define all outcomes for which data were sought. Specify whether all results that were compatible with each outcome domain in each study were sought (e.g. for all measures, time points, analyses), and if not, the methods used to decide which results to collect.	3-4
	10b	List and define all other variables for which data were sought (e.g. participant and intervention characteristics, funding sources). Describe any assumptions made about any missing or unclear information.	3-4
Study risk of bias assessment	11	Specify the methods used to assess risk of bias in the included studies, including details of the tool(s) used, how many reviewers assessed each study and whether they worked independently, and if applicable, details of automation tools	4

Section and Topic	Item #	Checklist item	Location where item is reported
		used in the process.	
Effect measures	12	Specify for each outcome the effect measure(s) (e.g. risk ratio, mean difference) used in the synthesis or presentation of results.	4
Synthesis methods	13a	Describe the processes used to decide which studies were eligible for each synthesis (e.g. tabulating the study intervention characteristics and comparing against the planned groups for each synthesis (item #5)).	5
	13b	Describe any methods required to prepare the data for presentation or synthesis, such as handling of missing summary statistics, or data conversions.	4
	13c	Describe any methods used to tabulate or visually display results of individual studies and syntheses.	4
	13d	Describe any methods used to synthesize results and provide a rationale for the choice(s). If meta-analysis was performed, describe the model(s), method(s) to identify the presence and extent of statistical heterogeneity, and software package(s) used.	4
	13e	Describe any methods used to explore possible causes of heterogeneity among study results (e.g. subgroup analysis, meta-regression).	4
	13f	Describe any sensitivity analyses conducted to assess robustness of the synthesized results.	4
Reporting bias assessment	14	Describe any methods used to assess risk of bias due to missing results in a synthesis (arising from reporting biases).	4
Certainty assessment	15	Describe any methods used to assess certainty (or confidence) in the body of evidence for an outcome.	4
**RESULTS**			
Study selection	16a	Describe the results of the search and selection process, from the number of records identified in the search to the number of studies included in the review, ideally using a flow diagram.	4 and Figure 1
	16b	Cite studies that might appear to meet the inclusion criteria, but which were excluded, and explain why they were excluded.	4 and Figure 1
Study characteristics	17	Cite each included study and present its characteristics.	4 and Tab1, Tab2, Tab3 and Tab4
Risk of bias in studies	18	Present assessments of risk of bias for each included study.	5-6
Results of individual studies	19	For all outcomes, present, for each study: (a) summary statistics for each group (where appropriate) and (b) an effect estimate and its precision (e.g. confidence/credible interval), ideally using structured tables or plots.	5-6 and Figure2, Figure3 and Figure4
Results of syntheses	20a	For each synthesis, briefly summarise the characteristics and risk of bias among contributing studies.	5-6

Section and Topic	Item #	Checklist item	Location where item is reported
	20b	Present results of all statistical syntheses conducted. If meta-analysis was done, present for each the summary estimate and its precision (e.g. confidence/credible interval) and measures of statistical heterogeneity. If comparing groups, describe the direction of the effect.	5-6
	20c	Present results of all investigations of possible causes of heterogeneity among study results.	5-6
	20d	Present results of all sensitivity analyses conducted to assess the robustness of the synthesized results.	5-6
Reporting biases	21	Present assessments of risk of bias due to missing results (arising from reporting biases) for each synthesis assessed.	5-6
Certainty of evidence	22	Present assessments of certainty (or confidence) in the body of evidence for each outcome assessed.	5-6
**DISCUSSION**			
Discussion	23a	Provide a general interpretation of the results in the context of other evidence.	14-15
	23b	Discuss any limitations of the evidence included in the review.	15
	23c	Discuss any limitations of the review processes used.	15
	23d	Discuss implications of the results for practice, policy, and future research.	16
**OTHER INFORMATION**			
Registration and protocol	24a	Provide registration information for the review, including register name and registration number, or state that the review was not registered.	3
	24b	Indicate where the review protocol can be accessed, or state that a protocol was not prepared.	3
	24c	Describe and explain any amendments to information provided at registration or in the protocol.	3
Support	25	Describe sources of financial or non-financial support for the review, and the role of the funders or sponsors in the review.	16
Competing interests	26	Declare any competing interests of review authors.	16
Availability of data, code and other materials	27	Report which of the following are publicly available and where they can be found: template data collection forms; data extracted from included studies; data used for all analyses; analytic code; any other materials used in the review.	NA

From: Page MJ, McKenzie JE, Bossuyt PM, Boutron I, Hoffmann TC, Mulrow CD, et al. The PRISMA 2020 statement: an updated guideline for reporting systematic reviews. BMJ 2021;372:n71. doi: 10.1136/bmj.n71

For more information, visit: http://www.prisma-statement.org/

Table S2. PECO (P = Population, E = Exposure, C = Comparison, O = Outcome) criteria for the systematic literature review on the association between alcohol intake and the incidence of fatal prostate cancer (PCa), PCa mortality, and the overall and cancer-specific survival, and clinical surrogates of survival, of PCa patients.

**Populations (P)**	Healthy subjects		PCa patients	
**Exposure (E)**	Current or former alcohol drinkers, or heavy drinkers, or subjects with a history of alcohol dependency / alcoholism.			
**Comparison (C)**	Never drinkers, or light drinkers, or subjects without a history of alcohol dependency / alcoholism.			
**Outcome (O)**	Fatal PCa incidence	PCa mortality	Overall and cancer-specific survival of PCa patients	Clinical surrogates of PCa patients' survival (e.g. biochemical failure and PSA growth)

Table S3: Main characteristics of the studies included in the review that reported on the association between alcohol intake and the risk of fatal prostate cancer.

First Author, PY	Period/ Follow-up	Country/ Study name	Study design	Study population	Alcohol assessment
Hsing A.W. et al. 1990 [28]	Baseline assessment 1966; last follow-up 1986 (data source: death certificates)	Lutheran Brotherhood Cohort Study	Cohort	n=17,633, median age 51 yrs	Questionnaire at baseline in 1966 (pre-diagnosis)
Platz A.E. et al. 2003 [29]	Baseline assessment 1986; last follow-up 1998 (data source: National Death Index and medical records)	US	Prospective cohort	n=47,843; range age=40-75 yrs	FFQ in 1986, 1988, 1990 and 1998
Watters L.J. et al. 2010 [30]	Baseline assessment 1995-1996; last follow-up 2005 (data source: national cancer registries)	US	Prospective cohort	n=294,707, range age=50-71 yrs	FFQ at baseline in 1995-1996 (pre-diagnosis)
		Swedish			not specified

Huynh-Le M.P. et al. 2021 [31]	Baseline assessment 1997; last follow-up 2011 (data source not specified)		Nestetd case-control study	n=2,163, median age=72.4 yrs	
Dahlman D. et al. 2022 [32]	Baseline assessment 1997; average follow-up time 15,2±5,6 yrs (data source: Swedish national register)	Swedish	Prospective cohort	n=1,361,532; range age 50-75 yrs	ICD-10 F10 and K7

Table S4: Main characteristics of the studies included in the review that reported on the association between alcohol intake and the risk of death from prostate cancer in healthy subjects.

First Author, PY	Period/ Follow-up	Country/ Study name	Study design	Study population	Alcohol assessment
Baglietto L. et al. 2006 [33]	Baseline assessment 1990-1994; last follow-up 2003 (data source: Victoria death records and National Death Index)	Australia	Prospective cohort	n=16,872; age range =27-75 yrs	Structured questionnaires and diaries at baseline (pre-diagnosis)
Kim M.K. et al 2010 [34]	Baseline assessment 2000; last follow-up 2005 (data source: national death certificate)	Korea	Prospective cohort	n=1,341,393; age range =40-69 yrs	Baseline assessment (pre-diagnosis); method not specified
Breslow R.A. et al. 2011 [35]	Baseline assessment 1988; last follow-up 2006 (data source: National Center for Health Statistics)	US	Prospective cohort	n=138,590; mean age=42.3 for "never drinker", 54.0 for "former drinker", 49.6 for "lifetime infrequent drinker"; 41. 7 for "light drinker", 42.5 for "moderate drinker", 41.4 for "heavier drinker"	Assessment at baseline in 1988 and later in 1990, 1991 and 1997-2004; method not specified
Fowke J.H. et al. 2015 [36]	Baseline assessment 1963; last follow-up 2006 (data source: death certificates)	Asia	PP[1] cohorts	n=522,736, mean age 53.7 yr	Questionnaire at baseline in 1963

Dickerman B.A. et al. 2016 [37]	Baseline assessment 1975; last follow-up 2012 (data source: Finnish Cancer Registry and Statistics Finland)	Finland	Prospective cohort	n=11,372; mean age=40.1 yrs	Questionnaires in 1975 and 1981

[1]PP;Prospective pooled

Table S5: Main characteristics of the studies included in the review that reported on the association between alcohol intake and the overall and cancer-specific survival of prostate cancer patients.

First Author, PY	Period/ Follow-up	Country/ Study name	Study design	Study population	Alcohol assessment
Yu G.P. et al. 1997 [38]	Incident cases of cancers histologically confirmed identified between 1990-1995 (data source: Cancer Data Base)	US	Prospective cohort	n=2,461; range age=0 to ≥70 yrs	Information obtained from the Cancer Data Base
Chamie K. et al. 2012 [39]	Baseline assessment 1997-2004; last follow-up 2010 (data source: California Cancer Registry)	California	Retrospective cohort	n=1,031; age range=66-75 yrs	Information obtained from medical records
Jayadevappa R. et al. 2016 [40]	Baseline assessment 2001-2004; follow-up 4 years after treatment phase (data source:National Cancer Institute)	US	Retrospective cohort	n=6,611; two age related sub-groups= "young-old aged 66-74 yrs, "old-old" aged ≥75 yrs	Three exclusive time-based categories of substance use as substance use in: pre-phase, treatment-phase, or follow-up phase according to International Classification of Diseases.
Brunner C. et al 2016 [41]	not specified	25 studies (US, Australia and European countries)	Mendelian Randomization study	n=23,868; age range= 58.4-72.1	not specified
Farris M.S. et al. 2018 [42]	Baseline assessment 1997-2000; last follow-up 2017 (data source: medical records)	Canada	Prospective cohort	n=829; mean age 67.3	In-person interviews (questionnaire, cognitive method and recall calendar) shortly after diagnosis and again 2-3 years post-diagnosis

Bluethmann S.M. et al. 2019 [43]	Prostate cancer survivors were identified between 2004-2014 (data source: Pennsylvania Cancer Registry)	Pennsylvania	Retrospective cohort	n=90,694; mean age= 66.19 (SD 9.25)	County-level estimates linking to individual-level data by county at time of PC diagnosis; aggregated 2011-2014 prevalence estimates (%) by behavioral risk factor surveillance system (BRFSS)
Downer M.K. et al. 2019 [13]	Baseline assessment 1986 until prostate cancer diagnosis or death; last follow-up 2012 (data source: medical records)	US	Prospective cohort	n=5,182; age range= 40-75 yrs	Food Frequency Questionnaires every 4 years

Table S6: Main characteristics of the studies included in the review that reported on the association between alcohol intake and clinical surrogates of survival of prostate cancer patients.

First Author, PY	Period/ Follow-up	Country/ Study name	Study design	Study population	Outcome	Alcohol assessment
Ly D. et al. 2010 [44]	Baseline assessment 1996, last follow-up 2005 (data source: prostate cancer registry at Cleveland Clinic)	Cleveland	Prospective cohort	n=2,687; median age 64 yrs	Biochemical Failure	Extracted from patient medical records
Burton A.J. et al. 2012 [45]	Baseline assessment 1999; last follow-up 2009 (mean duration of follow-up=4,77, SD 2.33)	UK	Prospective cohort	n=404, age range=50-69 yrs	PSA growth	Questionnaire (pre-diagnosis)

Table S7: Estimated alcohol intake espressed in grams per day (g/day) for estimates and studies included in the meta-analysis.

First Author, PY	GA	Comparison	Estimated comparison	RR (95%CI)	Adj
**Risk of fatal prostate cancer**					
Hsing A.W. et al. 1990 [28]	US	Beer Current[1] vs. never	Beer > 0.2g/day[1] vs. 0	**1.2 (0.8-1.7)**	Yes
Platz A.E. et al. 2003 [29]	US	Alcohol intake (g/day)[2] 30.0 -49.9 vs. 0	30.0 -49.9 g/day[2] vs. 0	Distant metastatic or fatal cases **1.09 (0.69-1.71)**	Yes
Watters L.J. et al. 2010 [30]	US	Alcohol (Drinks/Day)[3] ≥6 vs. 0	≥78g/day vs. 0[3]	Fatal cases **0.45 (0.25-0.81)**	Yes
Huynh-Le M.P. et al. 2021 [31]	Swedish	History of alcohol intake Yes vs. no	History of alcohol Intake Yes vs. 0	**1.44 (1.17-1.78)***	Yes
**Death from prostate cancer in healthy subjects**					
Baglietto L. et al. 2006 [33]	Australia	40+ g/day vs. Lifetime abstainers[4]	+40g/day vs. 0[4]	**0.73 (0.32,1.70)**	X
Kim M.K. et al 2010 [34]	Korea	≥90 g/day vs. Non drinker [5]	≥90 g/day vs. 0[5]	**2.39 (0.83-6.89)***	X
Breslow R.A. et al. 2011 [35]	US	Heavier[6] vs. Never drinker [7]	26g/day [6] vs. 0[7]	**0.89 (0.51-1.56)**	X
Fowke J.H. et al. 2015 [36]	Asia	≥156 g/week vs. 1-155 g/week [8]	≥22.3 g/day vs. 0.1-22.1 g/day [8]	**1.00 (0.74-1.35)**	X
Dickerman B.A. et al. 2016 [37]	Finland	Heavy vs. light drinkers[9]	>26g/day vs. 0.01–5.6 g/day[9]	**1.32 (0.66-2.62)**	X
**Cancer-specific survival of prostate cancer patients**					
Yu G.P. et al. 1997 [38]	US	Alcohol use: Ever vs. never	Ever vs. 0	**1.16 (0.70-2.0)**	
Farris M.S. et al. 2018 [42]	Canada	Post-diagnosis dose intake ≥8.3 drinks/week vs. none	≥15.4 g/day vs. 0	Overall survival: **0.90 (0.69-1.18)**	X

Bluethmann S.M. et al. 2019 [43]	US	Chronic drinking[10]<br>Subgroup by age:<br>40-54<br>55-64<br>65-74<br>75+	≥26 g/day[10] vs. 0<br>Subgroup by age:<br>40-54<br>55-64<br>65-74<br>75+	Cause-specific survival:<br>**0.80 (0.63-1.03)**<br>**1.00 (0.90-1.11)**<br>**0.95 (0.88-1.03)**<br>**1.03 (0.95-1.11)**	Yes
Downer M.K. et al. 2019 [13]	US	First post-diagnostic report intake ≥30g/day vs. none	≥30g/day vs. 0	Overall survival:<br>**0.76 (0.52-1.12)**	X

1 - Users: at least 6 times a year

2 - We multiplied servings of specified portions of each type of alcoholic beverage by grams of ethanol per serving (beer =12.8 g, red and white wine = 11.0 g, and liquor = 14.0 g) and summed the data for the different types of alcohol to obtain total alcohol intake in grams per day.

3 - One drink was defined as one 12-fluid-ounce beer, one 5-ounce glass of wine, or one 1.5-ounce shot of liquor, all equaling approximately 13 g of alcohol.

4 - Lifetime abstaines <12 alcoholic drinks in a year

5 - Non-drinker, 1.0–14.9, 15.0–29.9, 30.0–89.9, ≥90.0 g/day for men; Non-drinker, 1.0–14.9, ≥15.0 g/day for women.

7 - Never drinker if they had also consumed fewer than 12 drinks over the course of their lifetime.

6 - Heavier drinker: women >7 drinks per week; men >14 drinks per week;

8 Alcohol consumption was calculated in grams per week to unify data on alcohol intake across studies, assuming standard serving sizes (beer = 355 mL, wine = 125 mL, spirits = 35 mL), a standard portion of ethanol per drink (beer = 5%, wine = 12%, spirits = 40%), and 0.789 g of ethanol per mL.

9 Light drinker (average of 0.01–3 drinks/week), moderate drinker (average of 3.01–14 drinks/week), and heavy drinker (average of >14 drinks/week) – defining one drink as 12 grams of alcohol.

10 - Chronic use of alcohol/high risk for heavy drinking (defined as an average ≥2 drinks per day for the last 30 days vs. <2 drinks).

Tables S8. Quality in Prognostic Studies (QUIPS) tool

Study (First author, year)	QUIPS domains					
	a) Study participation	b) Study attrition	c) Prognostic factor measurement	d) Outcome measurement	e) Study confounding	f) Statistical analysis and reporting
**Studies reporting on overall and cancer-specific survival of prostate cancer patients**						
**Yu, 1997**	moderate bias	moderate bias**	low bias	moderate bias	high bias	high bias
**Chamie, 2012**	low bias	NA	moderate bias	low bias	low bias	low bias
**Jayadevappa, 2016**	low bias	NA	low bias	low bias	low bias	low bias
**Brunner, 2016**	moderate bias	NA	moderate bias	moderate bias	moderate bias	low bias
**Farris, 2018**	low bias	low bias	low bias	low bias	low bias	low bias
**Bluethmann, 2019**	low bias	NA	low bias	low bias	low bias	low bias
**Downer, 2018**	low bias	moderate bias*	low bias	low bias	low bias	low bias
**Studies reporting on surrogates of survival survival of prostate cancer patients**						
**Ly, 2010**	low bias	moderate bias**	low bias	low bias	high bias	high bias
**Burton, 2012**	low bias	moderate bias**	low bias	low bias	low bias	low bias

*Mean follow-up rates exceed 90% but there is no description of individuals who dropped out of the study
** Despite being prospective studies, if they do not report precise information on this item

Table S9. Newcastle-Ottawa quality assessment scale

Newcastle-Ottawa quality assessment scale	Studies about incidence of fatal prostate cancer					Studies about prostate cancer mortality				
	Hsing, 1990	Platz, 2003	Watters, 2010	Huynh-Le, 2021	Dahlman, 2022	Baglietto, 2006	Kim, 2010	Breslow, 2011	Fowke, 2015	Dickerman, 2016
**Selection**										
1) Representativeness of the exposed cohort	0	0	1	1	1	1	1	1	1	1
2) Selection of the non-exposed cohort	1	1	1	1	1	1	1	1	1	1
3) Ascertainment of exposure	1	1	1	0	1	1	1	1	1	1
4) Demonstration that outcome of interest was not present at start of study	0	1	1	0	1	1	1	0	1	1
**Comparability**										
1) Comparability of cohorts on the basis of the design or analysis	1	2	2	1	2	2	2	2	2	2
**Outcome**										
1) Assessment of outcome	1	1	1	0	1	1	1	1	1	1
2) Was follow-up long enough for outcomes to occur	1	1	1	1	1	1	0	1	0	1
3) Adequacy of follow-up of cohorts	1	1	0	0	0	1	0	0	0	1
**Total**	**6**	**8**	**8**	**4**	6	**9**	**7**	**7**	**7**	**9**

Table S10. PRISMA 2020 for Abstracts

Section and Topic	Item #	Checklist item	Reported (Yes/No)
**TITLE**			
Title	1	Identify the report as a systematic review.	Yes
**BACKGROUND**			
Objectives	2	Provide an explicit statement of the main objective(s) or question(s) the review addresses.	Yes
**METHODS**			
Eligibility criteria	3	Specify the inclusion and exclusion criteria for the review.	No
Information sources	4	Specify the information sources (e.g. databases, registers) used to identify studies and the date when each was last searched.	Yes
Risk of bias	5	Specify the methods used to assess risk of bias in the included studies.	No
Synthesis of results	6	Specify the methods used to present and synthesise results.	Yes
**RESULTS**			
Included studies	7	Give the total number of included studies and participants and summarise relevant characteristics of studies.	Yes
Synthesis of results	8	Present results for main outcomes, preferably indicating the number of included studies and participants for each. If meta-analysis was done, report the summary estimate and confidence/credible interval. If comparing groups, indicate the direction of the effect (i.e. which group is favoured).	Yes
**DISCUSSION**			
Limitations of evidence	9	Provide a brief summary of the limitations of the evidence included in the review (e.g. study risk of bias, inconsistency and imprecision).	Yes
Interpretation	10	Provide a general interpretation of the results and important implications.	Yes
**OTHER**			
Funding	11	Specify the primary source of funding for the review.	No
Registration	12	Provide the register name and registration number.	No
